# Supplementary material for: Stromal and epithelial transcriptional map of initiation progression and metastatic potential of human prostate cancer
Source: Nat Commun. 2017 Sep 4;8:420. doi: 10.1038/s41467-017-00460-4 (PMC5583238; doi:10.1038/s41467-017-00460-4)
Supplement: Supplementary file 1 — Supplementary Information [file 41467_2017_460_MOESM1_ESM.pdf]

File Name: Supplementary Information
Description: Supplementary Figures

File Name: Supplementary Data 1
Description: Genes differentially expressed between Gleason 6 and 8+ tumor epithelium and surrounding stroma.

File Name: Supplementary Data 2
Description: GO biological processes significantly overrepresented at 0.05 FDR in 24 gene stromal gene signature.

File Name: Peer Review File
Description:

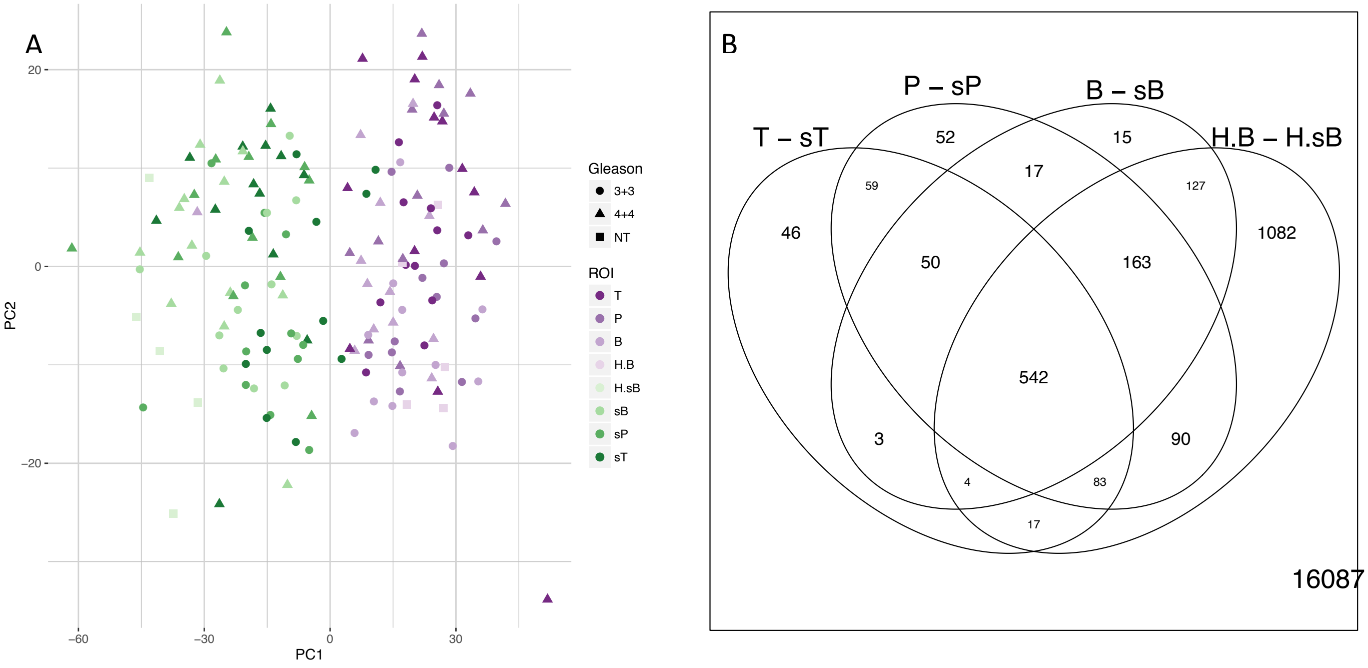

Supplementary Figure 1 (A) Principal components plot showing clear separation of the epithelial and stromal compartment on the first principal component (B) Venn diagram showing gene associations from the 4 epithelial-stroma comparisons between the three morphologically distinction regions.

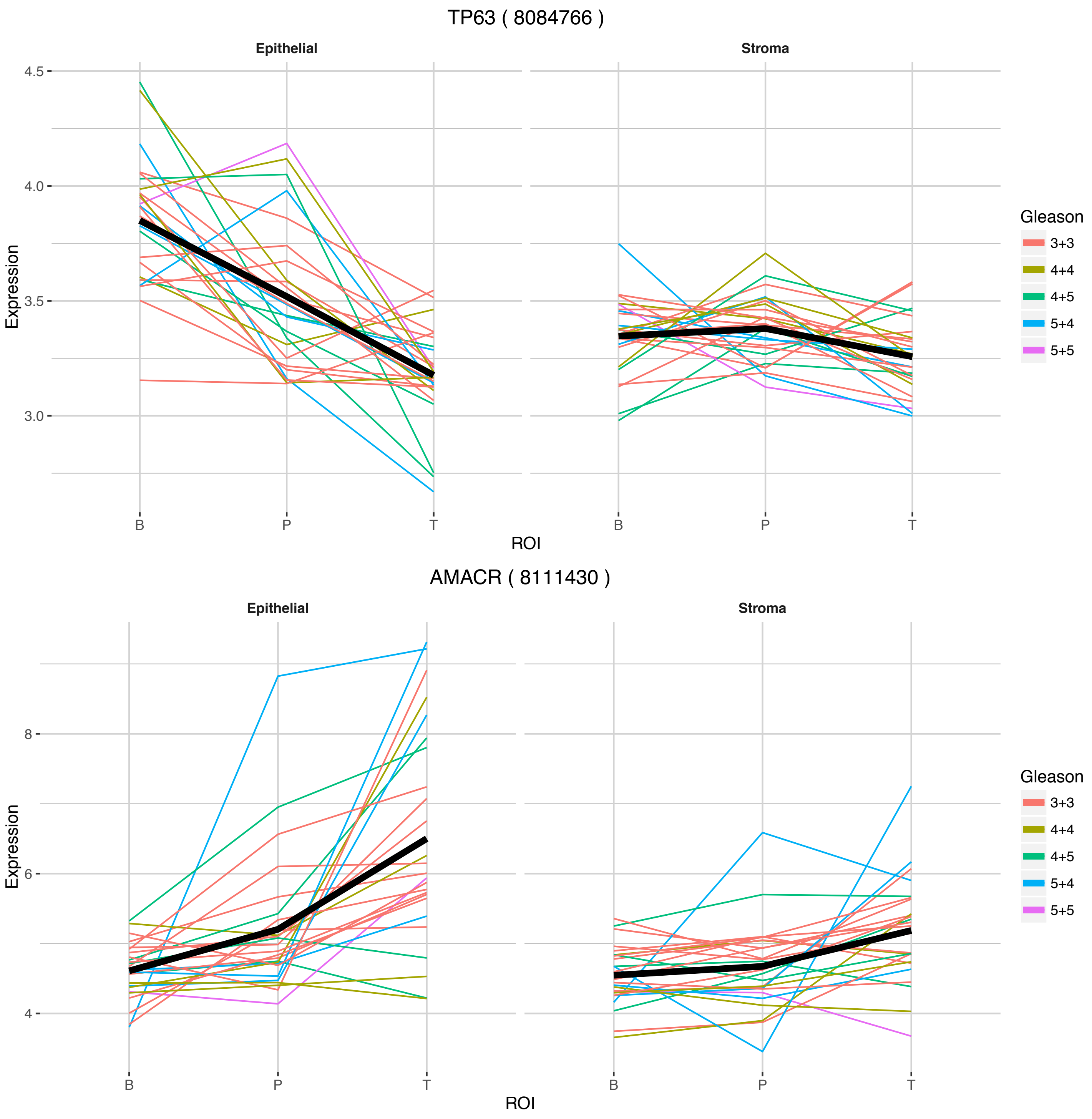

Supplementary Figure 2 Gene expression of TP63 and AMACR across ROI in epithelium and stroma.

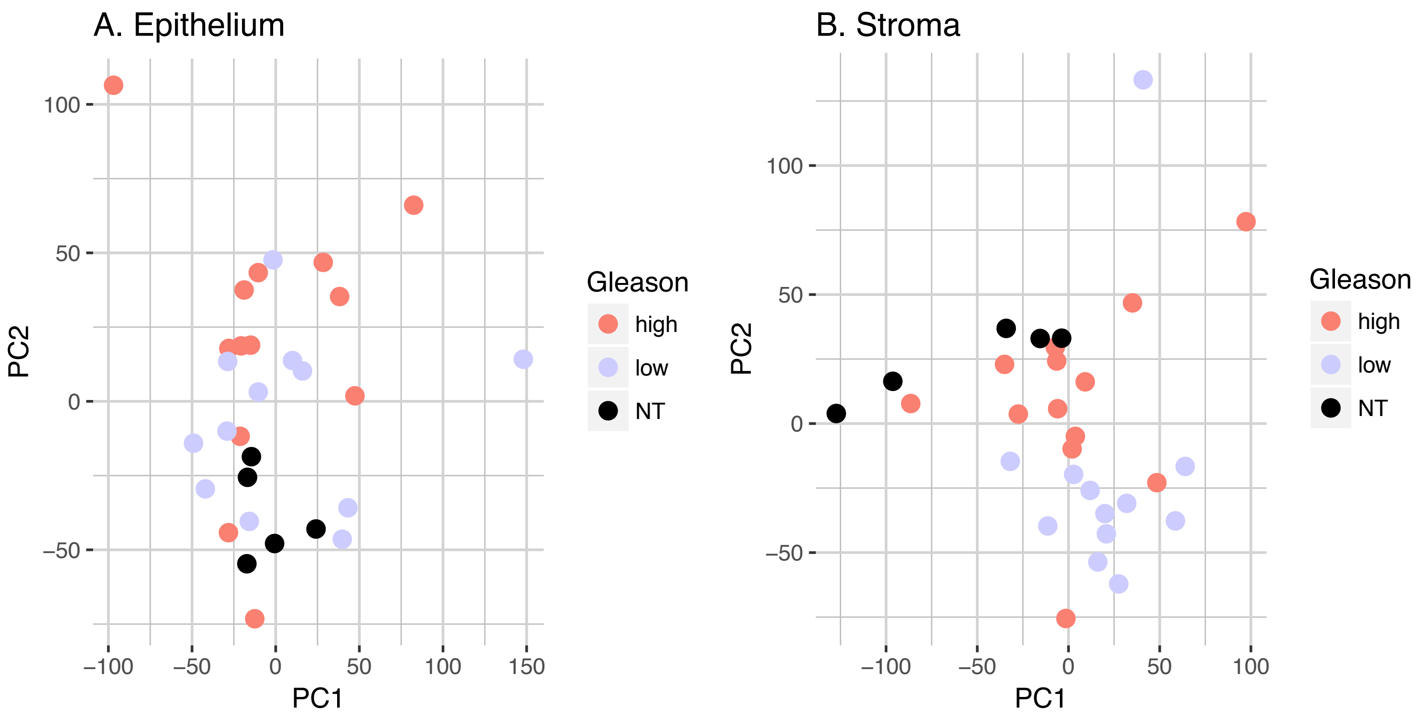

Supplementary Figure 3 Principal components plots for (A) benign epithelium and (B) benign stroma from cystoprostatectomy and RP cases. Color of the points reflect 3 groups of samples: NT - benign tissue from cystoprostatectomy patients that had no tumor, "high" and "low" – benign tissue from RP patients that had high and low Gleason grade disease respectively. While gene expression seems to be more homogeneous for epithelial cells, expression in the stroma seems to be more structured, with benign stroma from RPs with high grade tumors being intermediate between stroma of RPs with low grade tumors and cystoprostatectomy cases with no tumors.

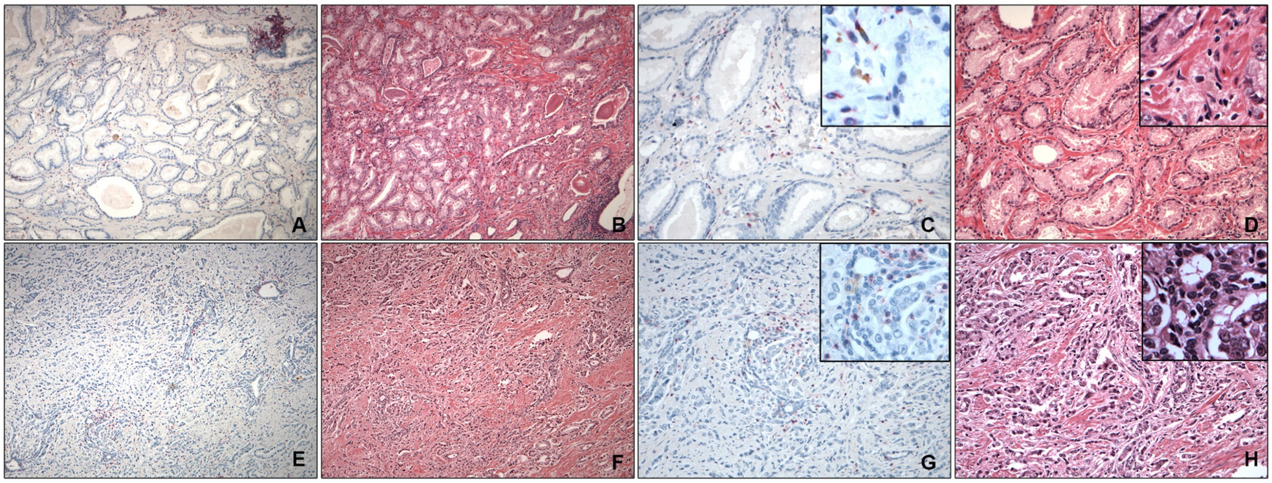

Supplementary Figure 4 Representative immunohistochemistry for CD45 (red) and CD163 (brown) for the assessment of lymphocyte and macrophage infiltration in invasive tumors, with corresponding H&E stains. (A) Low power view of a low Gleason grade case; (B) its corresponding H&E. (C) High power view of stromal area of the same low grade case showing scant lymphocytic infiltrate (red) and very rare macrophages (brown), seen better in the inset; (D) its corresponding H&E. (E) Low power view of a high Gleason grade case; (F) its corresponding H&E; (G) high power view of stromal area of the same high grade case showing scant lymphocytic infiltrate (red) and very rare macrophages (brown), seen better in the inset; (H) corresponding H&E. Note on panel (A) of the figure a cluster of lymphocytes around normal glands (top right hand corner). Very consistently these infiltrates are seen in the adjacent normal tissue and never in the tumor.

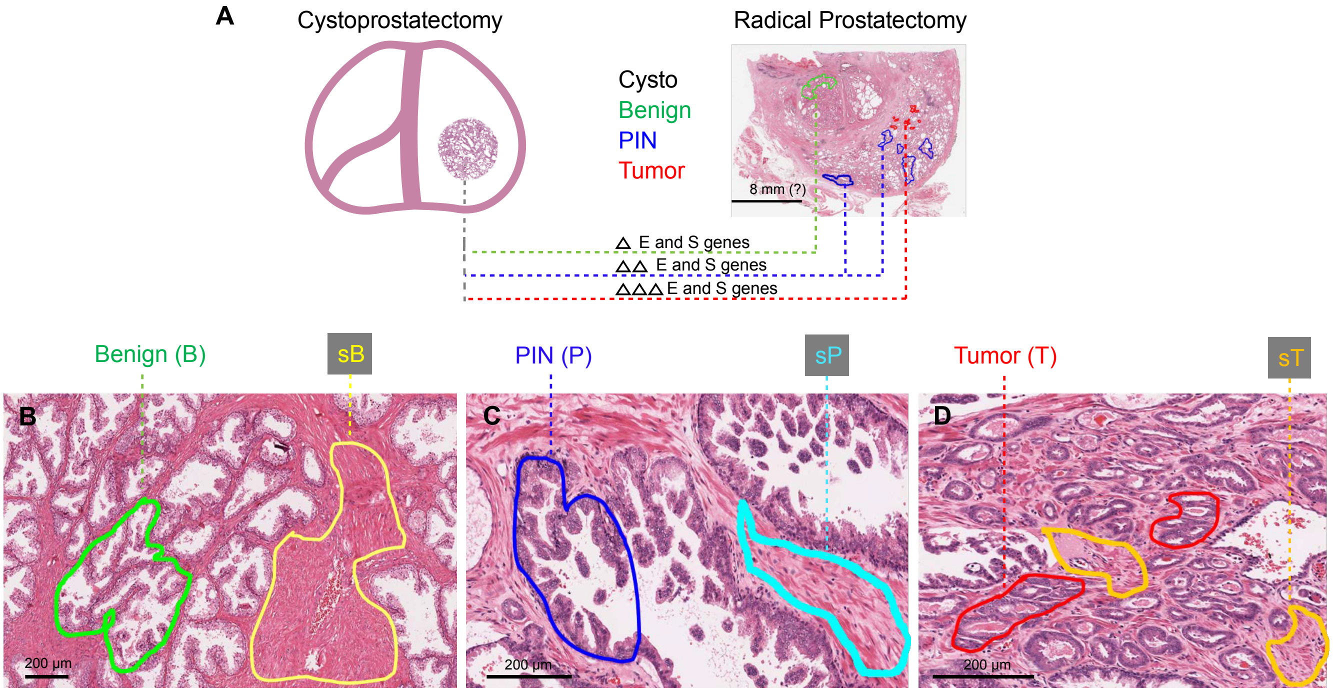

Supplementary Figure 5 (A)Representative image of the circled H&E slide with all annotate regions of interest. Red circling indicates tumor, blue hgPIN and green benign regions of interest used for subsequent microdissection. (B-D) Higher magnification images of the representative images of Benign, PIN and Tumor epithelial tissue and their adjacent stroma.
